# Renal cell carcinoma with gallbladder metastasis: a case report

**DOI:** 10.11604/pamj.2022.43.106.36588

**Published:** 2022-10-27

**Authors:** Hamza Dergamoun, Abdelaziz El Gdaouni, Imad Ziouziou

**Affiliations:** 1 Department of Anatomy, Agadir University Hospital, Faculty of Medicine and Pharmacy, Ibn Zohr University, Agadir, Morocco,; 2 Department of Urology, Agadir University Hospital, Faculty of Medicine and Pharmacy, Ibn Zohr University, Agadir, Morocco

**Keywords:** Renal cell carcinoma, gallbladder, metastasis, case report

## Abstract

Renal cell carcinoma (RCC) is the most frequent renal neoplasm, with a high rate of metastasis, especially in the lungs and bones. The gallbladder is one of the rare metastatic sites. We report an 80-year-old woman who presented with chronic right flank pain for the last six months. A computer tomography scan revealed a heterogeneous right renal mass measuring 86 ×76× 68 mm and multi lithiasis in the gallbladder. A right radical nephrectomy, lymphadenectomy, and cholecystectomy were performed. The postoperative clinical course was uneventful, without any complications. The histological results showed a clear RCC with metastasis to the gallbladder. After 12-months follow-up, the patient is free from disease. In conclusion, even though the coexistence of metastatic gallbladder from clear RCC is rare, the possibility of concurrence should be considered if suspected findings in the gallbladder are identified intraoperatively.

## Introduction

Renal cell carcinoma (RCC) accounts for approximately 90% of renal neoplasms, with a high rate of metastasis [1]. The most common metastatic sites are lungs (75%) and bones (20%) [1]. At the time of the diagnosis, 30% of patients with RCC have one or multiple metastases [2,3]. The gallbladder is one of the very rare metastatic sites. It was found in only 0.58% of autopsy cases of RCC [3]. There are sparse reports of RCC and gallbladder coexistence in the literature [4-6]. Because of its rarity, the clinicopathological feature of gallbladder metastasis from RCC remains poorly understood, and the challenge for the surgeon is the decision about whether to remove the gallbladder in a patient diagnosed with RCC with concurrent gallbladder anomaly that does not meet the traditional indications for cholecystectomy. In this paper, we report an exceptional case of RCC with gallbladder metastasis. We also discuss the clinical features and the specificities of therapeutic management of this rare condition.

## Patient and observation

**Patient information:** an 80-year-old woman was admitted for chronic right flank pain in the last six months, with one episode of hematuria. The patient mentioned a history of weight loss, decreased appetite, and generalized weakness. There was no significant medical or family history or any relevant past interventions.

**Clinical findings:** the physical examination was unremarkable. The temperature was 37.3°C, blood pressure 124/84 mmHg, and pulse rate regular at 86 beats/min. In palpation, we revealed mild tenderness in the right flank but without any palpable mass.

**Figure 1:**
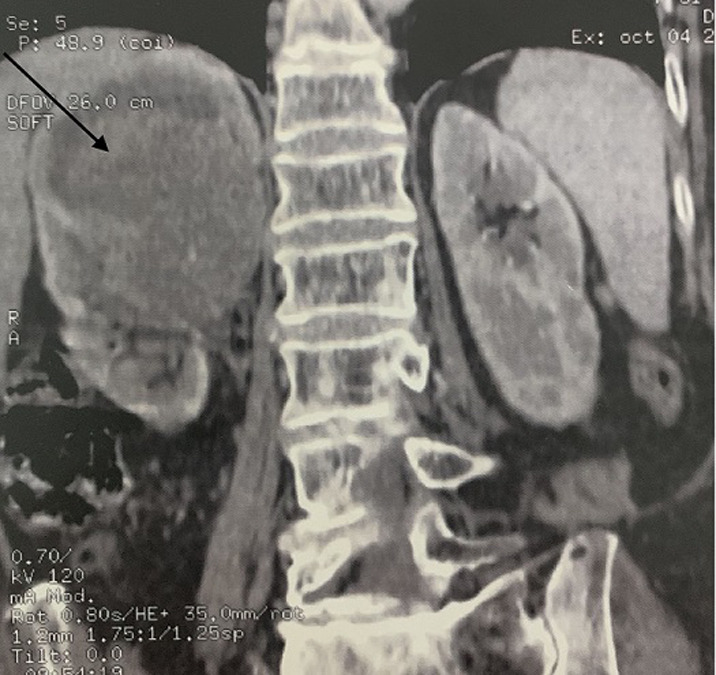
abdominal computed tomography scan showing a mass in the right kidney 86 x 76 x 68 mm

**Figure 2:**
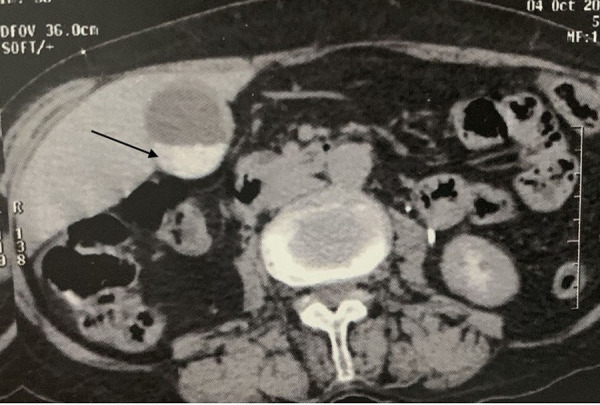
abdominal computed tomography scan showing a gallstone

**Diagnostic assessment:** abdominal computer tomography (CT) scan revealed a heterogeneous right renal mass measuring 86 x76x 68 mm and arising from the upper and middle pole (Figure 1), with a lymph node of 8 mm diameter detected in the renal hilum as well as a multi lithiasis gallbladder (Figure 2). Laboratory tests did not detect any alterations, and gave the following results: white blood cell count was 79.000/mm^3^, red blood cell count 5,3.106/mm^3^, hemoglobin 13.0 g/dL, hematocrit 39.1%, platelets 19.6 x 104/µL, total bilirubin 0.6 mg/dL, direct bilirubin 0.2 mg/dL, aspartate transaminase 25 IU/L, alanine transaminase 19 IU/L, albumin 4.25 g/ dL, lactate dehydrogenase 240 IU/L, β-glutamyltransferase 27 IU/L, alkaline phosphatase 201 IU/L, amylase 129 IU/L, blood urea nitrogen 11.6 mg/dL, creatinine 8 mg/L, C-reactive protein 3 mg/L, urinalysis pH 7.0, no uric protein, no urinary sugar, no ketone body, no uric blood, no bilirubin, and no white blood cell, and no bacteriuria. A chest CT was done and did not show any metastasis. The presumptive diagnosis was a renal tumor classified T2a-N2-M0, associated with a multi lithiasis gallbladder.

**Therapeutic interventions:** a right open radical nephrectomy via subcostal incision with lymph node dissection and cholecystectomy was performed. The surgical exploration showed a right renal large superior polar exophytic mass with two palpable hilar lymphadenopathies. The wall of the gallbladder appeared normal except for its lithiasis content. The surgical intervention was performed successfully without adhesion or local invasion.

**Figure 3:**
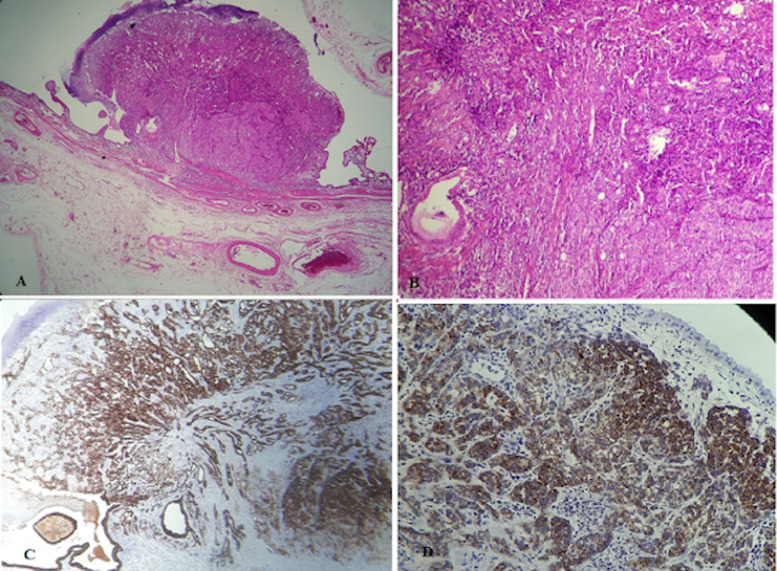
histological examination images for the gallbladder: (A) gallbladder wall showing tubullopapillary carcinoma, low power x 25; (B) gallbladder wall showing tubullopapillary carcinoma, high power x 100; (C) immunohistochemistry findings: CK7 positive; (D) immunohistochemistry findings: AMACR-positive

**Follow-up and outcome of interventions:** the postoperative course was without complications, and the patient was uneventfully discharged on the fifth postoperative day. Pathological assessment of the specimen showed the tumor was well-circumscribed, measuring 9 x 7 x 5 cm and subtotally effacing the kidney. At sectioning, it was tan-yellow with large necrotic areas. The gallbladder was thin-walled with a 0.4 x 0.3 submucosal nodule. Microscopically the renal tumor consisted of a tubulopapillar lined by pseudostratified layers of cells with abundant eosinophilic cytoplasm and high nucleolar grade. It was a clear cell RCC with the pathologic stage of pT2a, Fuhrman 3. The polypoid lesion of the gallbladder was identical to those of the renal tumor population, made up of large, plant-like cells with clear cytoplasm, adopting an acinar or solid architecture within a stroma rich in small vessels (Figure 3A, Figure 3B). Immunohistochemistry was positive for cytokeratin CK7, AMACR (a-Methylacyl Coenzyme A Racemase), cytokeratin (AE1/AE3), and vimentin (Figure 3C, Figure 3D). The morphologic and phenotypic profiles were consistent with synchronous metastasis from renal carcinoma. Additionally, the histopathological study found several lymph nodes to be invaded. So, the final stage of the tumor was pT2a-N2-M1. The follow-up was carried out by CT scan at six months, and then after one year, the patient did not present a recurrence, and the renal function remained normal.

**Patient perspective:** the patient was informed about the all procedure, complication and outcome, and she was agreeing about it. The patient was pleased with the care she received throughout therapy.

**Informed consent:** written informed consent was obtained from the patient for participation in our study.

## Discussion

Kidney cancers account for 4% of all newly diagnosed malignancies in men, and most of these are RCCs [2,4]. About 30% of RCCs cases have metastases at diagnosis [4-6]. The typical sites of metastases are the lungs, brain, liver, and lymph nodes, but they are also known to have rare metastasis [7]. The gallbladder is not among the usual sites of RCC metastases, and they are reported only sporadically in case of reports [4-6]; this metastasis is often metachronous, with a median interval of four years after nephrectomy [8]. The clear cell type was responsible for practically all cases of metastasis of RCC to the gallbladder. It is still unclear if this is due to the fact that clear cell RCC is the most frequent type of RCC or whether this type of metastasis is exclusive to clear cell type [9].

The gallbladder metastasis of RCC was often asymptomatic and discovered on imaging or autopsy [7,10], but some can present with biliary colic or acute cholecystitis, and acute cholecystitis as a clinical presentation is thought to be associated with a poor prognosis [3,5]. A recent literature review mentioned a male predominance with infections occurring at a 2/1 male to female ratio and a median age of diagnosis at 62 years old [4]. In our case, the patient had no biliary symptoms, and the preoperative imaging revealed no gallbladder abnormalities. Previous studies have shown that gallbladder metastases are not associated with gallstones, whereas primary cholangiocarcinoma is frequently associated with lithiasis disease [3]. However, in the present case, a nodule-mimicking gallstone was diagnosed during laparotomy, which turned out to be a malignant metastasis of the RCC. The rarity of such presentation, female gender, older age, and the unremarkable medical history of our patient together made the diagnosis extremely challenging. Despite the variety of imaging modalities available, it can be challenging to distinguish neoplastic and non-neoplastic gallbladder polyps due to their significant overlap in appearance. Transabdominal ultrasound is good for detecting gallbladder polyps, but it has a poor ability to distinguish between true and pseudo polyps [11]. It may also report signs to differentiate a metastatic tumor from a primary, while a metastatic tumor may be accompanied by a hyperechoic band on the surface of the tumor, whereas a primary tumor often protrudes into the lumen of the gallbladder, being large at the wall, hypoechoic and presenting an inhomogeneous echogenic pattern [5].

CT also plays an important role; a CT study by Cho *et al*. [11] analyzed the imaging characteristics of gallbladder metastases and reported that the characteristic pattern of gallbladder metastases from RCC is the appearance of polypoid lesions with strong enhancement in the early and late arterial phase and washout in the portal phase [11], whereas primary gallbladder cancer does not show a hypervascular pattern [5]. However, for small polyps measuring <1 cm, imaging studies are limited in their ability to identify features associated with malignancy [11]. In the present study, we used immunohistochemical analysis to distinguish between primary gallbladder cancer and metastasis of RCC. In general, primary gallbladder carcinoma consistently shows positive staining for CK7, EMA, and CEA but is negative for CA9 and vimentin [12]. On the contrary, metastatic clear cell RCC shows the opposite staining pattern [12]; these immunohistochemical findings might help in the differential diagnosis.

There is no definitive treatment strategy for gallbladder metastasis of RCC [13]. Most patients with gallbladder metastasis of RCC at diagnosis have a widespread disease with several metastases in other organs, and their prognosis is poor [3]. However, in fewer cases, the gallbladder is the unique location of metastasis [13]. The clinical practice guideline for RCC in Japan reviewed that metastasectomy is expected to improve survival for RCC patients if they have good performance status and a long disease-free period, and complete resection is possible [12]. In this context, simple cholecystectomy may be enough to completely remove the gallbladder metastasis located and would be sufficient treatment for the management of the metastasis of the gallbladder with a primary RCC [13], leading to a better outcome and survival, like our patient [7]. The recurrence rate in the recent literature review was mentioned as about 48% [4], while Chung et al. conducted a study following 33 patients and ultimately found a five-year survival rate of 35%-50% following the procedure, and the authors mentioned that the prognosis was significantly decreased if multiple metastatic sites had occurred [14]. Adjuvant therapies following cholecystectomy, including chemotherapy, radiotherapy, and immunotherapy, have been proposed, but there is no consensus regarding their roles [8].

## Conclusion

Even though the coexistence of RCC and gallbladder metastasis is of rare occurrence, the surgeon should consider the possibility of synchronous of those tumors if any incidental mass is identified during surgery. Metastatic gallbladder tumor is very difficult to diagnose because it is easily mistaken for benign disease during common radiologic imaging studies. Notwithstanding the few data in the literature, surgery for metastatic gallbladder disease of renal origin seems to be a feasible treatment and capable of increasing patients’ overall survival. Further studies should focus on how to identify diagnosis and on effective other treatment options.
